# Potential Mechanisms of Action for Vitamin C in Cancer: Reviewing the Evidence

**DOI:** 10.3389/fphys.2018.00809

**Published:** 2018-07-03

**Authors:** Margreet C. M. Vissers, Andrew B. Das

**Affiliations:** Centre for Free Radical Research, Department of Pathology and Biomedical Science, University of Otago, Christchurch, Christchurch, New Zealand

**Keywords:** ascorbate, hypoxia-inducible factor, hydrogen peroxide, iron-mediated autoxidation, antioxidant, epigenetic demethylases, ten-eleven translocases, HIF hydroxylases

## Abstract

Whether vitamin C (ascorbate) has a role to play as an anti-cancer agent has been debated for decades. Ascorbate has been used by cancer patients in an unregulated environment, either as a dietary supplement or in pharmacological doses administered by infusion, with numerous reports of clinical benefit, but in the absence of rigorous clinical trial data. The design of appropriate clinical trials has been hindered by a lack of understanding of the mechanism(s) of action that would inform the choice of effective dose, timing of administration and likely responsive cancer models. More recently, expanded understanding of the biological activities of ascorbate has led to a number of plausible hypotheses for mechanisms of anti-cancer activity. Prominent among these are the generation of significant quantities of hydrogen peroxide by the autoxidation of supra-physiological concentrations of ascorbate and stimulation of the 2-oxoglutarate-dependent dioxygenase family of enzymes (2-OGDDs) that have a cofactor requirement for ascorbate. Hydrogen peroxide generation is postulated to generate oxidative stress that preferentially targets cancer cells. The 2-OGDDs include the hydroxylases that regulate the hypoxic response, a major driver of tumor survival, angiogenesis, stem cell phenotype and metastasis, and the epigenetic histone and DNA demethylases. The latter are of particular interest, with recent studies suggesting a promising role for ascorbate in the regulation of the ten-eleven translocase (TET) DNA demethylases in hematological cancers. Support for these proposed mechanisms has come from many *in vitro* studies, and xenograft animal models have consistently shown an anti-cancer effect of ascorbate administration. However, decisive evidence for any particular mechanism(s) of action is not yet available from an *in vivo* setting. With a number of early phase clinical trials currently underway, evidence for potential mechanism(s) of action is required to inform the most appropriate study design and choice of cancer model. Hopefully such information will result in sound clinical data that will avert adding any further controversy to this already contentious debate.

## Introduction

Whether vitamin C has a role to play in the development and regulation of cancer growth has been a topic of investigation and discussion for decades. There is a substantial body of literature that documents potential anti-tumor effects of ascorbate in *in vitro* and *in vivo* settings, with many reporting cytotoxicity toward cancer cells and a slowing of tumor growth in animal models (for reviews, see Du et al., 2012; Park, 2013; Fritz et al., 2014; Wilson et al., 2014; Mata et al., 2016; Cimmino et al., 2018; Klimant et al., 2018). Human clinical studies, however, have been infrequent, with most recent phase I/II studies aiming to determine the tolerability of pharmacological doses of ascorbate for patients with advanced cancer (Hoffer et al., 2008, 2015; Stephenson et al., 2013). Some of these studies have suggested that high dose ascorbate treatment may have a clinical benefit for patients with pancreatic cancer (Monti et al., 2012; Cieslak and Cullen, 2015) and other advanced cancers (Hoffer et al., 2015) but too few patients have been analyzed in these studies to date. In addition, the use of dietary vitamin C supplements has been linked to improved patient outcome in breast cancer (Harris et al., 2013, 2014).

Interest in the use of ascorbate for cancer was initiated in the 1970’s when Pauling and Cameron advocated for its use following their observations of apparently extended survival in patients with advanced cancer treated with intravenous infusions of ∼10 g ascorbate daily (Cameron and Pauling, 1976, 1978). At the time, these claims were met with incredulity by many, mostly because the available information on the biological activity of ascorbate could not be reconciled with a plausible anti-cancer mechanism. There were proposals that ascorbate deficiency might interfere with the synthesis of connective tissues, particularly collagen (McCormick, 1959; Cameron and Pauling, 1973), but there was no explanation that could explain the need for intravenous administration, as deficiency could be easily overcome by dietary supplementation. The Mayo Clinic attempted to repeat Pauling and Cameron’s results in a randomized placebo-controlled study in patients with advanced cancer, but they failed to detect any clinical benefit after daily oral administration of 10 g of vitamin C (Moertel et al., 1985). The fundamental difference between the two data sets is now believed to be the oral versus intravenous route of ascorbate, which is known to result in significantly altered pharmacokinetics of the vitamin (Padayatty and Levine, 2001; Padayatty et al., 2004). When viewed alongside a more sophisticated understanding of tumor biology, this information of the pharmacokinetics and functions of vitamin C has led to the development of new hypotheses for potential mechanisms of anti-cancer activity. This crucial information was unavailable in the 1970’s, but has now engendered renewed consideration of the potential role for ascorbate in cancer.

High dose ascorbate infusions are an easily accessed therapy used by both medical practitioners and complementary and alternative medicine providers, but without robust evidence of clinical efficacy (Padayatty et al., 2010). There is an acknowledged need for good clinical studies to determine the anticancer efficacy of this practice and to establish appropriate clinical guidelines. Case reports suggest that there may be circumstances under which vitamin C can provide a clinical benefit (these are usually high intravenous doses, given as a course over months) (Drisko et al., 2003; Padayatty et al., 2006; Mikirova et al., 2016; Raymond et al., 2016) and recent phase I trials have indicated that high-dose vitamin C is a useful adjunct to chemotherapy (Welsh et al., 2013; Hoffer et al., 2015). However, given the prevalence of ascorbate use by cancer patients, clinical advantage is not commonly seen, suggesting that only a subset of patients may benefit. This highlights the problem: how to identify the potentially responsive patients and determine the best treatment regimen, dose and frequency of intervention? To address these questions, it is essential that we understand how ascorbate might act in cancer. Currently a number of hypotheses are under investigation and in this review we will consider the evidence to date in support of these, evaluated against what we know about ascorbate chemistry and biology.

## Uptake and Metabolism of Ascorbate

Ascorbate is a small, highly water-soluble molecule derived from glucose and present in all plants and animals (Hornig, 1981; Smirnoff and Wheeler, 2000; Padayatty and Levine, 2016). Most animals synthesize it in the liver or kidneys but it has become a vitamin for humans, other primates, guinea pigs and fruit bats, which have mutations in gulonolactone oxidase, the terminal enzyme in the biosynthetic pathway (Chatterjee et al., 1975; Banhegyi et al., 1997; Linster and Van Schaftingen, 2007; Du et al., 2012). In all animals, ascorbate is transported to the tissues via the circulation and most cells concentrate the vitamin to levels many-fold above plasma levels (average ∼50 μM) through active uptake by the sodium-dependent vitamin C transporters SVCT1 and SVCT2 (Tsao, 1997; May and Qu, 2005; Savini et al., 2008; Harrison and May, 2009; Mandl et al., 2009; Nualart et al., 2014). An exception to this is the red blood cell, which does not express SVCT2 and accumulates ascorbate by uptake of dehydroascorbate (DHA) via the GLUTs (Tu et al., 2017). Tissue levels vary considerably, with some organs, notably brain, adrenals, liver, and white cells containing concentrations up to 20 mM (Tsao, 1997). High intracellular levels are thought to reflect a demand for ascorbate as an essential enzyme cofactor (Tsao, 1997; May and Qu, 2005).

The high water-solubility and active transport of ascorbate in the body means that this compound is readily acquired and distributed, but not stored, and turnover is constant. The reliance on dietary intake in humans can therefore lead to deficiency if adequate intake is not maintained and this is pertinent to the effects of ascorbate supplementation in cancer. Turnover appears to be accelerated during periods of illness (Bonham et al., 1999; Fain et al., 2003; Gan et al., 2008; Evans-Olders et al., 2010) and, although information is sparse, many cancer patients are found to be ascorbate deficient when measurements have been made (Anthony and Schorah, 1982; Ray and Husain, 2001; Mayland et al., 2005; Shah et al., 2009; Badid et al., 2010; Nagamma et al., 2014). These low plasma levels are likely to correlate with lower tissue levels; this has been demonstrated in white blood cells (Levine et al., 2001) and in a mouse model of ascorbate dependency low plasma levels resulted in almost complete tissue deficiency (Vissers et al., 2011). In contrast, animals that synthesize ascorbate are known to maintain plasma saturation levels regardless of health challenges, dramatically increasing production to compensate for increased turnover when ill (Chatterjee et al., 1975; Michels and Frei, 2013; Campbell et al., 2015). This is an important point for consideration in experiments using animal tumor xenograft models. Unless the study is carried out with guinea pigs or a mutant animal such as the Gulo^-/-^ knock-out mouse, the plasma and tissue ascorbate levels can be maintained at a high baseline level by endogenous synthesis and supplementation may have little impact. Wild-type animals, and knock-out animals bred onto a wild-type background such as the Gulo^-/-^ mouse, also demonstrate a limited capacity for ascorbate uptake through the gut (Michels and Frei, 2013). Hence, if these animals are used in an experimental setting, dietary supplementation may have limited effect, and plasma and tissue levels should be monitored throughout to confirm ascorbate status and the applicability of the model for the human condition. It should be noted that such measurements are rarely carried out, and this can complicate the interpretation of published experimental data.

## The Chemistry and Functions of Ascorbate

The chemistry of ascorbate dictates its biological activity. It is possibly best known for its capacity to act as an antioxidant: this property reflects its ability to readily undergo one- or two-electron oxidation, generating the relatively stable ascorbyl radical or DHA, respectively. DHA is unstable at neutral pH, and unless it is reduced *in vivo* by glutathione or thioredoxin to regenerate reduced ascorbate, it will rapidly decompose to diketogulonic, oxalic and threonic acids (Washburn and Wells, 1999; Smirnoff, 2000), which are eliminated from the body via the kidneys (Washko et al., 1992, 1993; Washburn and Wells, 1999; Linster and Van Schaftingen, 2007). Consequently, DHA represents only a very small fraction of the ascorbate present *in vivo* and is not likely to be present at concentrations above 1–2 μM (Dhariwal et al., 1991; Michels and Frei, 2013; Pullar et al., 2018). The amount present *in vivo* may often be overestimated when blood or tissues are analyzed, as it seems that most of the DHA detected is likely to reflect oxidation in air during sample processing (Dhariwal et al., 1991; Levine et al., 1998; Michels and Frei, 2013; Pullar et al., 2018). The oxidation of ascorbate and breakdown of DHA is likely to account for daily turnover, and this may be exacerbated during periods of illness (Chatterjee et al., 1975; Bonham et al., 1999; Fain et al., 2003; Gan et al., 2008; Evans-Olders et al., 2010).

**FIGURE 1:**
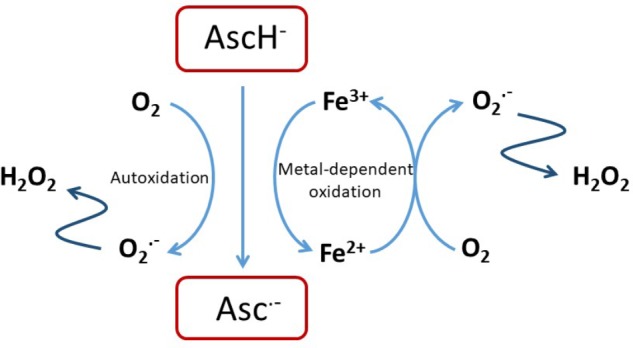
Pro-oxidant activity of ascorbate. Reactions of ascorbate with oxygen or free transition metal ions such as Fe^2+/3+^ or Cu^+/2+^ can result in the generation of H_2_O_2_ that is itself cytotoxic of that can undergo further reaction to exacerbate oxidative stress (Shah et al., 2009; Vissers et al., 2011; Nualart et al., 2014).

Ascorbate is able to chelate and reduce transition metal ions, particularly Fe^3+^ and Cu^2+^ (Du et al., 2012; Padayatty and Levine, 2016), and this property contributes to its ability to promote iron uptake from the diet (Lane and Richardson, 2014). It also enables a pro-oxidant activity through the recycling of Fe^2+^ (Eq. 1), and in the presence of oxygen leads to the generation of superoxide, H_2_O_2_ and highly reactive oxidants such as the hydroxyl radical by promoting the Fenton reaction (Eq. 2) and Haber–Weiss chemistry (Winterbourn, 1981; Buettner, 1987; Smirnoff, 2000; Koppenol, 2001; Levine et al., 2011; Michels and Frei, 2013) (**Figure 1**).

(1) $$\begin{array}{c} {\mathrm{AscH}}^{–}{+Fe}^{3}{}^{+}\to {\mathrm{Asc}}^{•–}{+Fe}^{2}{}^{+}{+H}^{+} \end{array}$$

(2) $$\begin{array}{c} {\mathrm{Fe}}^{2}{}^{+}{+H}_{2}O_{2}{+H}^{+}\to {\mathrm{Fe}}^{3}{}^{+}{+H}_{2}{O+HO}^{•}\; \end{array}$$

The ability to reduce transition metals is likely to be fundamental to the activity of ascorbate as an essential co-factor for many Fe- and Cu-containing enzymes; in this capacity it ensures that enzyme activity is optimized by the maintenance of the active-site metals in the reduced state (Kivirikko et al., 1989; Rebouche, 1991; Ozer and Bruick, 2007; Mandl et al., 2009; Grosso et al., 2013; Vissers et al., 2014).

## Anti-Cancer Activities of Ascorbate

All the known properties of ascorbate are being considered for the investigation of its potential anti-cancer activity. There are numerous studies that have demonstrated a cytotoxic effect of ascorbate on tumor cells *in vitro*, either alone or in combination with chemotherapeutics (Wells et al., 1995; Reddy et al., 2001; Pathak et al., 2002; Wozniak and Anuszewska, 2002; Guerriero et al., 2006; Kassouf et al., 2006; Chen et al., 2007, 2012; Martinotti et al., 2011; Verrax et al., 2011; Ma et al., 2014; Cieslak et al., 2015; Xia et al., 2017) or radiation (Herst et al., 2012; Castro et al., 2014). The cytotoxicity in many of these studies reflects the oxidative stress resulting from the H_2_O_2_ generated in cell culture medium when ascorbate is present at concentrations of 1 mM or above, and manifests as increased cell cycle arrest, p53 upregulation, decreased ATP levels, compromised mitochondrial function, suppression of antioxidant gene expression NrF-2 and/or cell death by apoptosis (Tarumoto et al., 2004; Fromberg et al., 2011; Rouleau et al., 2016; Yang et al., 2017). Anti-cancer effects have also been demonstrated with ascorbate levels well below 1 mM: levels as low as 100 μM or even 1 μM in the culture medium enhanced the susceptibility of cancer cells to etoposide, cisplatin, or doxorubicin (Kurbacher et al., 1996; Reddy et al., 2001; Tarumoto et al., 2004; An et al., 2011). The mechanism of action at these low concentrations remains unclear, but potentially involves modification of cell survival pathways involving p53 (Kurbacher et al., 1996; Reddy et al., 2001; Tarumoto et al., 2004; An et al., 2011). The highly variable experimental designs and differences in outcome in these *in vitro* studies highlight the complex nature of investigations involving the addition of ascorbate to cell culture systems, with both artifacts and actual cell functional changes being possible. The interactions of ascorbate with chemotherapeutics are likely to reflect the redox properties of extracellular ascorbate and the many intracellular co-factor activities. There is also a clinical concern that ascorbate could impede the action of chemotherapy drugs (Ludke et al., 2009, 2010; Perrone et al., 2009), but this area remains poorly understood. There is significant consensus that ascorbate can act synergistically with gemcitabine (Kassouf et al., 2006; Espey et al., 2011; Martinotti et al., 2011; Volta et al., 2013; Cieslak et al., 2015), and treatment with a combination of the two agents did not result in any adverse events in recent phase I/II trials (Monti et al., 2012; Welsh et al., 2013).

**FIGURE 2:**
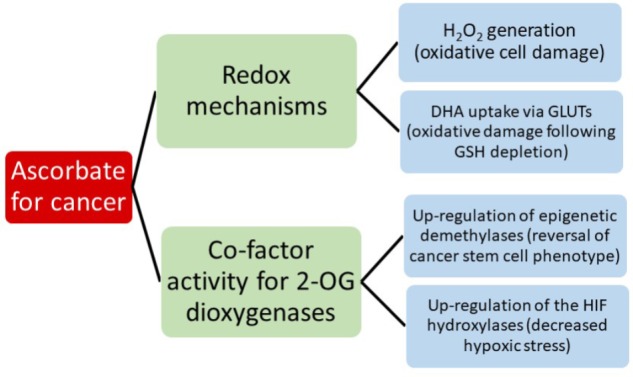
A summary of the current hypotheses that may contribute to anti-cancer activity by ascorbate.

Consideration of all the potential interactions between ascorbate and chemotherapeutics is beyond the scope of this review, but it should be noted that a clear understanding of this area will be important when considering introducing ascorbate into a clinical cancer setting. Instead, we aim to consider the evidence for the most current hypotheses that suggest that the anti-cancer activity of ascorbate reflects either its redox, pro-oxidant or enzyme co-factor activity (**Figure 2**). A significant body of literature is emerging that tests these ideas and we will consider the extent to which the current evidence supports these mechanisms.

### Mouse Xenograft Models

The use of mouse tumor xenograft models is common to many investigations, and there is a high degree of consistency in the reported results: administration of supplemental ascorbate, either through the diet or via high dose peritoneal administration, results in a decreased growth rate with a range of different tumors (Gao et al., 2007; Chen et al., 2008; Cha et al., 2011, 2013; Espey et al., 2011; Ma et al., 2014; Campbell et al., 2015, 2016b; Yun et al., 2015). The similarity of the data despite differences in tumor models and dosage regimes for ascorbate has reinforced support for ascorbate having an anti-cancer function, but in most cases the mechanism of action is inferred from data obtained *in vitro*. Very few studies have reported changes in the tumor biology that allow insight into the mechanism of action and conclusions beyond a simple correlation between tumor growth and ascorbate availability. Without this information, the *in vivo* data from animal studies cannot directly test a working hypothesis and this will be considered below.

### Ascorbate as a Pro-oxidant

The realization that intravenous ascorbate infusion results in plasma concentrations in the mM range, albeit transiently (half-life ∼2 h) (Padayatty et al., 2004), led to the suggestion that autoxidation of ascorbate and/or the stimulation of metal-catalyzed production of reactive oxygen species could generate significant quantities of H_2_O_2_ that are selectively toxic to cancer cells (Carosio et al., 2007; Belin et al., 2009). In tissue culture media, ascorbate concentrations above 1 mM are known to be cytotoxic (Buettner, 1988; Miller et al., 1990; Halliwell et al., 2000; Chen et al., 2005, 2007, 2008). The rate of ascorbate autoxidation is slowed when iron chelators are added to the medium and cytotoxicity can be inhibited by catalase, supporting the dependency of this phenomenon on the generation of H_2_O_2_ (Halliwell et al., 2000; Clement et al., 2001; Wee et al., 2003; Chen et al., 2007). Cancer cells have been shown to be more susceptible to H_2_O_2_ and oxidative stress than primary cell lines which is related to the metabolism of H_2_O_2_ and antioxidant capacity (Doskey et al., 2010; Du et al., 2010; Erudaitius et al., 2010; Olney et al., 2013; Schoenfeld et al., 2017). In contrast to tissue culture media, autoxidation of ascorbate is slow in plasma and at neutral pH (Buettner, 1988), but the ascorbyl radical has been detected in biological fluids that can contain traces of catalytic Fe (Buettner and Chamulitrat, 1990; Chen et al., 2007).

As indicated above, ascorbate-mediated cytotoxicity toward cancer cells *in vitro* has been carried out with concentrations in excess of the 1 mM threshold that induces significant H_2_O_2_ (Chen et al., 2011; Espey et al., 2011; Fromberg et al., 2011; Rouleau et al., 2016; Kim et al., 2018). These observations have fuelled the pilot clinical studies and phase I clinical trials involving the intravenous administration of pharmacological doses of ascorbate that aim to achieve mM levels in the circulation and extracellular fluid of the tumor (Hoffer et al., 2008, 2015; Monti et al., 2012; Stephenson et al., 2013; Welsh et al., 2013). While there are suggestions of a clinical benefit from these vitamin C infusions (Hoffer et al., 2008, 2015; Stephenson et al., 2013; Welsh et al., 2013), it remains a challenge to determine whether this is due to an ascorbate-mediated pro-oxidant effect. It is unclear whether H_2_O_2_ is generated in sufficient concentrations in a tumor *in vivo* to damage the cancer cells. Given that the autoxidation of ascorbate is highly dependent on the availability of oxygen, there is a question around the impact of the hypoxic tumor environment on the generation of H_2_O_2_. To our knowledge, definitive evidence for H_2_O_2_-mediated cytotoxicity initiated by supra-physiological ascorbate concentrations *in vivo*, such as detection of oxidative damage in tumors exposed to high dose ascorbate *in situ*, is not yet available.

### Cancer Cell Uptake of DHA and Associated Oxidative Stress

Dehydroascorbate is structurally similar to glucose and can be taken up into cells via the GLUTs and this may contribute to the intracellular pool in red blood cells, in neutrophils at sites of infection and in other regions of the body (Rumsey et al., 1997, 2000; Corpe et al., 2005; Wilson, 2005; Michels and Frei, 2013). Once inside the cell, DHA is reduced by GSH, NADH, and NADPH-dependent enzymes, thereby potentially depleting the cell of these crucial molecules (May et al., 1997, 1999; May, 2002, 2011). A connection between this phenomenon and the upregulation of GLUT1 in *KRAS* and *BRAF* mutant cells was recently proposed to account for the anti-tumor activity of ascorbate in colorectal cancer (Yun et al., 2015). *KRAS* and *BRAF* mutations are common in colorectal cancer and are associated with upregulation of GLUT1 and a glycolytic phenotype. Cultured cancer cells harboring mutations in *KRAS* and *BRAF* were shown to take up DHA via GLUT1 when incubated with mM concentrations of ascorbate, and this was associated with a loss of cell viability. The results were extrapolated into an *in vivo* mouse model with implanted mutant *KRAS* and *BRAF* cancer cells in which tumor growth was slowed when the animals were administered high doses of vitamin C by daily intraperitoneal injection of 4 g/kg. The data in this comprehensive study clearly show that the upregulated expression of GLUT1 can result in rapid uptake of DHA by these cancer cell lines, but there is much less certainty about the causal relationship between this and the observed cytotoxicity. At the 1–2 mM concentrations of ascorbate used, the generation of H_2_O_2_ in the medium via autoxidation, as discussed above, could equally result in depletion of GSH and cytotoxicity. This possibility could be eliminated by including a H_2_O_2_ scavenger such as catalase in the culture medium, and without this information, it remains uncertain whether cell death resulted from the uptake of DHA and subsequent intracellular redox stress, as was proposed, or whether it was due to H_2_O_2_ generated in the medium. Whether *KRAS*-mutant tumor growth inhibition in the vitamin C-treated mice was related to markers of oxidative stress in the tumor was not tested. Higher levels of ascorbate, and fewer intestinal polyps, were reported in *Kras^G12D^* mice treated with tamoxifen than in mice without this mutation, but whether these polyps showed higher levels of oxidative stress was not determined. Metabolic markers of glycolysis were decreased in *KRAS* and *BRAF* mutant cells in culture, and although this may be consistent with the hypothesis under investigation, it could equally be effected by downregulation of a hypoxic phenotype or by H_2_O_2_-mediated toxicity.

As previously discussed, DHA is unstable at neutral pH and is generally not detected in plasma or tissue samples (Dhariwal et al., 1991; Michels and Frei, 2013; Pullar et al., 2018). Therefore, when considering the likelihood of DHA uptake via the GLUTs contributing to the anti-tumor activity of ascorbate, it will be important to demonstrate that DHA exists *in vivo* in sufficiently high concentrations to be able to compete with physiological concentrations of glucose. Interestingly, ascorbate was recently reported to enhance the cytotoxicity of cetuximab in *KRAS* mutant colon cancer cell lines and co-incidentally reverse the glycolytic Warburg phenotype (Aguilera et al., 2016). Also, in another study, this activity was associated with the cellular accumulation of ascorbate in an SVCT2-dependent manner (Jung et al., 2016). Together, these studies paint a complex picture of the interaction of ascorbate with colon cancer cells.

### Downregulation of the Hypoxic Phenotype

Rapid cell division and poor blood vessel formation in a developing tumor result in local areas of oxygen and nutrient deprivation, leading to activation of the HIFs. These transcription factors determine the successful tumor adaptation to a stressful microenvironment, driving the transcription of numerous genes involved in glycolysis and glucose transport, angiogenesis, metastasis and resistance to chemo- and radio-therapy (Semenza, 2010, 2016; Ratcliffe, 2013). High HIF activity has been shown to promote the expression of a stem cell phenotype in breast cancer (De Francesco et al., 2015; Semenza, 2015, 2016) and is associated with a poor prognosis in a number of cancers (Dachs and Tozer, 2000; Vleugel et al., 2005; Cao et al., 2009; Semenza, 2010, 2015, 2016; Volinia et al., 2012; Wang et al., 2012, 2014; Zhang et al., 2012; Deb et al., 2014; Liu et al., 2014; Li et al., 2016; Schoning et al., 2017). Consequently the HIFs are now considered an important target for cancer therapy.

Activation of the HIFs is controlled by proline and asparaginyl hydroxylases (the HIF hydroxylases) that modify the regulatory HIF-α subunit (Bishop and Ratcliffe, 2014; Pugh and Ratcliffe, 2017), targeting the protein for proteasomal degradation and preventing the formation of an active transcriptional complex. The HIF hydroxylases belong to the family of iron-containing dioxygenases that utilize oxygen and 2-oxoglutarate as substrates (Ozer and Bruick, 2007), and for which ascorbate functions as a co-factor, possibly by maintaining the active site Fe in the reduced state (Koivunen et al., 2004). The co-factor role is specific to ascorbate (Myllyla et al., 1978), which is structurally specific for the hydroxylase active site (Kaczmarek et al., 2009). When cells are deficient in ascorbate, HIF hydroxylase activity is compromised and HIF transcription activity is increased, particularly in response to conditions of mild or moderate hypoxia (Knowles et al., 2003; Vissers et al., 2007; Kuiper et al., 2014a; Campbell et al., 2016a). These observations have led to the hypothesis that increasing ascorbate supply to cancer cells could stimulate the activity of the HIF hydroxylases and decrease the activation of the HIFs, thereby slowing tumor growth rates.

There is a growing body of evidence consistent with this hypothesis. Investigations with the vitamin C-dependent Gulo^-/-^ mouse have indicated a strong association between tumor ascorbate content, HIF activation and tumor growth *in vivo* following variable dietary intake or intra-peritoneal injection of pharmacological ascorbate (Campbell et al., 2015, 2016a). The daily administration of high dose vitamin C to tumor-bearing Gulo^-/-^ mice indicated that HIF-1 transcriptional activity was depressed in association with ascorbate uptake. Elevation of tumor ascorbate content was transient following intraperitoneal infusion and levels were maintained with daily, but not alternate day, administration. Decreased HIF-1 expression and slowed tumor growth also required daily administration and ascorbate levels were associated with reduced tumor microvessel density and decreased regions of hypoxia, suggesting more effective oxygen delivery throughout the tumor (Campbell et al., 2016b).

Retrospective analysis of human tumor tissue has identified an inverse association between the amount of ascorbate present in the tumor and markers of HIF activation in endometrial, colorectal and thyroid cancer tissue (Kuiper et al., 2010, 2014b; Jozwiak et al., 2015). Higher tumor ascorbate was associated with increased disease-free survival for colorectal cancer patients (Kuiper et al., 2014b). Taken together, these data provide strong evidence for an ascorbate-mediated downregulation of HIF transcriptional activity that would be consistent with slower tumor growth.

### Epigenetic Mechanisms of Action

Genetic and epigenetic changes are inextricably linked with the development of cancer (Baylin and Jones, 2011, 2016). One of the most striking epigenetic changes in cancer is global DNA hypomethylation, which can result in genomic instability and increased chromosomal fragility (Berman et al., 2011; Ehrlich and Lacey, 2013). Furthermore, hypomethylation can activate the transcription of transposable elements and oncogenes, providing multiple mechanisms by which it can contribute to oncogenesis. In addition to global hypomethylation, hypermethylation localized to the promotors of tumor suppressor genes has also been established (Baylin and Jones, 2011). In contrast, most gene promotors that contain CpG islands are not methylated in adult stem cells, and this is crucial for these genes to remain active or ready to be activated (Shen and Laird, 2013). These findings are encouraging for the development of cancer therapies because epigenetic changes are reversible, whereas the cancer-driving mutations themselves are much more difficult to target and revert.

The role of ascorbate in epigenetics has received increasing attention in multiple contexts, from the normal functioning of cells to the treatment of cancer (Lorsbach et al., 2003; Monfort and Wutz, 2013; Young et al., 2015; Camarena and Wang, 2016; Gillberg et al., 2017; Cimmino et al., 2018; Mastrangelo et al., 2018). Of particular relevance to this discussion is the direct involvement of the 2-OGDDs in epigenetic regulation, specifically the ten-eleven translocase (TET) and JMJC families. The TET family was named after the initial discovery of TET1 as a translocation partner of MLL in AML with *t*(10;11)(q22;q23) (Ono et al., 2002; Lorsbach et al., 2003). Subsequent homology searches in mammalian genomes detected two other members of this family, TET2 and TET3, which along with TET1 catalyze the conversion of 5-methylcytosine (5mC) to 5-hydroxymethylcytosine (5hmC) (Tahiliani et al., 2009). Further oxidation of 5-hmC to 5-formylcytosine (5fC) and then 5-carboxylcytosine (5caC) is also carried out by the TET proteins. Collectively, these oxidative steps lead to both active and passive demethylation of DNA (He et al., 2011; Kohli and Zhang, 2013). The JMJC family contains more than 20 proteins that collectively are able to demethylate mono-, di-, and tri- methylated histone lysine residues (Tsukada et al., 2006; Monfort and Wutz, 2013). Each demethylation step involves the oxidation of a methyl group, followed by the spontaneous removal of formaldehyde. These discoveries have established the TET and JMJC dioxygenases as integral components for active demethylation of DNA and histones, respectively.

Altered expression or mutations affecting these dioxygenases have been detected in solid tumors from a wide range of tissues as well as in hematological malignancies (Gillberg et al., 2017). Given that ascorbate is required for optimal activity of both families (Klose et al., 2006; Tsukada et al., 2006; Minor et al., 2013) and that that the majority of mutations in these cancers affect one copy of the gene, it is possible that ascorbate could compensate by increasing the remaining enzyme activity. The majority of evidence supporting this mechanism comes from models of AML. Next generation sequencing of large cohorts of patients with AML have shown that approximately 10% of patients carry a mutation in TET2 that mostly affects only one allele (Ley et al., 2013; Papaemmanuil et al., 2016). JMJC mutations are much less frequent with changes to KDM5A and KDM6A each detected in ∼1% of patients. Interestingly, mutations in IDH are mutually exclusive with TET2, with IDH1 or IDH2 affected in approximately 20% of cases. The reason for this exclusivity demonstrates the strong link between metabolism and epigenetics. IDH converts isocitrate to 2-OG (a required cofactor for TET and JmjC enzymes). In contrast, mutant IDH (mutIDH) generates 2-hydroxyglutarate (2-HG) instead, which has been shown to impair hematopoiesis by inhibiting TET2 (Figueroa et al., 2010; Losman et al., 2013).

Building on this information, three recent studies have explored the potential of ascorbate to restore normal cell function in mouse and cellular models of leukemia involving either mutant TET2 or IDH (Agathocleous et al., 2017; Cimmino et al., 2017; Shenoy et al., 2017). Cimmino et al. (2017) used a transgenic mouse model whereby TET2 knockdown was inducible and reversible using RNAi. They showed that TET2 knockdown resulted in aberrant self-renewal, loss of myeloid lineage markers, significant hypermethylation, and decreased transcription of genes that are hypermethylated in AML patients. Both TET2 restoration and ascorbate (250 μM) reversed these changes. It is interesting to note that the addition of catalase (1 unit/mL of medium) had no effect on ascorbate’s ability to reverse aberrant self-renewal in re-plating assays. Furthermore, ascorbate at this concentration did not increase intracellular dichlorofluorescein fluorescence, an indicator of intracellular reactive oxygen species, which could be detected with as little as 50 μM H_2_O_2_ (Wang et al., 2014).

Agathocleous et al. (2017) used a number of different mouse models including various combinations of *Tet2* deficiency, *Gulo^-/-^*, and *Flt3^ITD^* (an activating mutation that cooperates with *Tet2* loss to induce AML). Firstly, they found that human and mouse HSCs had unusually high ascorbate levels that correlated with expression levels of the ascorbate transporter *Slc23a2*. Secondly, they showed that *Gulo^-/-^* mice had increased number HSC numbers that was in part due to decreased TET2 activity. In an elegant series of experiments involving transplantation of *Flt3^ITD^* donor bone marrow cells into either wild-type or *Gulo^-/-^* recipients, they showed that ascorbate deficiency was able to cooperate with *Flt3^ITD^* to promote leukemogenesis in a manner similar to *Tet2* loss. Furthermore, the development of myeloid leukemia was accelerated when *Tet2^Δ/+^*;*Flt3^ITD^* donor bone marrow cells were transplanted into *Gulo^-/-^* recipients with respect to wild-type recipients. Finally, supplementation of a 1% ascorbate diet (Harlan) to *Tet2^Δ/+^*;*Flt3^ITD^*;*Gulo^-/-^*mice at 7 weeks, significantly prolonged survival compared to those that were not supplemented. Related experiments using *Tet2^Δ/Δ;^Flt3^ITD^* donor bone marrow cells showed that these effects were mediate via *Tet2* dependent as well as *Tet2* independent mechanisms. It is possible that *Tet2* independent effects are mediated via *Tet1* or *Tet3*, but this will need to be explored in future experiments.

The third study utilized HOXA9-immortalized mouse bone marrow cells expressing *IDH1^*R132H*^* in order to explore the effects of ascorbate treatment (Mingay et al., 2018). Ascorbate treatment (345 μM) promoted DNA demethylation at enhancers that are implicated in myeloid differentiation. Ascorbate treatment also resulted in the promotor demethylation and increased expression of several key hematopoietic genes. It is important to note that 2-phosphate ascorbic acid was used for cell culture experiments. This was done in order to explicitly avoid the extracellular production of H_2_O_2_.

Cumulatively, these data suggest that ascorbate is able to mitigate the effect of *Tet2* loss in models of leukemia at least in part by upregulating TET2 activity. Given the low concentrations of ascorbate used, it is unlikely that the effects seen are mediated via the production of H_2_O_2_. These findings warrant the investigation of using ascorbate to improve TET activity in patients with heterozygous *TET* or *IDH* mutations. This is particularly relevant for AML, where *TET2* and *IDH* mutations are early drivers (Papaemmanuil et al., 2016). *TET2* mutations occur in the white blood cells of otherwise healthy adults with clonal hematopoiesis (Busque et al., 2012), and it is conceivable that ascorbate supplementation could prevent progression to myelodysplastic syndrome or overt AML. One recent clinical trial in AML patients looked at ascorbate supplementation as an adjunct to DNA methyltransferase inhibition (DNMTi) therapy in elderly patients aged 60–87 (Zhao et al., 2018). They compared ascorbate plus low-dose decitabine prior to aclarubicin and cytarabine (A-DCAG, *n* = 39) vs. DCAG alone (*n* = 34). Clinical remission was significantly higher after one round of treatment in the ascorbate group, which was associated with a higher overall survival. Unfortunately, no data was provided with regards to *TET2* mutation status in the patient group. Future clinical trials involving ascorbate will need to stratify response by mutation status if epigenetic mechanisms of action are to be validated in patient cohorts.

## Pharmacokinetic Considerations for Ascorbate in Cancer

The hypotheses discussed above all require adequate access for ascorbate to the tumor cells via effective distribution throughout the tumor environment. An understanding of the pharmacokinetics of ascorbate delivery into the extracellular tumor space and uptake into the cells is essential to make sense of the impact of ascorbate administration and to design effective treatment protocols for clinical intervention studies. As noted above, there is a fundamental difference between the plasma pharmacokinetics after oral or intravenous administration (Padayatty et al., 2004). Modeling the profile of plasma levels following intravenous infusion predicted that a peak concentration in the mM range would be achievable, and this has subsequently been measured in a number of studies (Hoffer et al., 2008; Robitaille et al., 2009; Stephenson et al., 2013). In tumor-bearing Gulo^-/-^ mice, elevated ascorbate levels were seen to persist in the tumor for more than 24 h post administration of high dose intraperitoneal injection, and this was attributed to increased stability in the hypoxic tumor environment (Campbell et al., 2016b).

From all current evidence, including animal studies and the information available from the available human clinical studies, there does seem to be an advantage for intravenous infusion for cancer, but optimal protocols for dosage and frequency of administration have not been developed. A dose of ∼1 g/kg is often administered to cancer patients (Padayatty et al., 2010) and is apparently well tolerated with minimal side-effects (Stephenson et al., 2013). That the frequency of ascorbate administration may also be important is suggested by the finding that the anti-tumor activity of ascorbate was more effective following daily administration to Gulo^-/-^ mice than when the infusions were given on alternate days (Campbell et al., 2016b).

**FIGURE 3:**
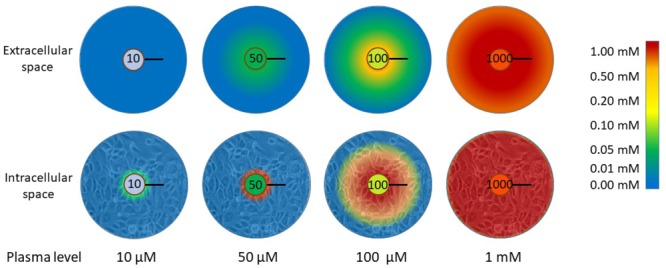
The distribution of ascorbate through extracellular tissue and into tissue cells in relation to plasma concentration. When plasma levels are low (10 μM), virtually no ascorbate is able to reach the tissues. This represents a state close to scurvy (complete deficiency). There is a significant difference in the capacity for ascorbate to accumulate in tissue cells when plasma levels are in the ‘healthy but not saturated’ range (50 μM) or saturated (100 μM). Below saturation, only cells adjacent to the blood vessel wall accumulate physiological intracellular concentrations (low mM). Diffusion of ascorbate does not extend beyond 100 μm with plasma ascorbate ≤100 μM and cells beyond this limited distance cannot accumulate ascorbate. To reach beyond this diffusion limit, supra-physiological levels are required. The diffusion distance for oxygen in tissues is 100 μm and this is the average distance between blood vessels in well-perfused tissues (Ludke et al., 2009, 2010; Volta et al., 2013). The data represented in this figure are from Kuiper et al. (2014c). The black bars represent 100 μm.

Tumor cells, as all tissue cells, are dependent on the plasma supply for nutrient delivery. The poor vasculature in tumors prevents effective oxygen delivery and drives the upregulation of the hypoxic response (Ratcliffe, 2013; Semenza, 2016). Oxygen has a diffusion distance of around 100 μm in tissues and this coincides with the inter-vessel distance in well-perfused normal tissues (Ratcliffe, 2013; Semenza, 2016). It is possible, therefore, that ascorbate delivery is similarly compromised when the vasculature is dysfunctional and disorganized. When the diffusion of ascorbate through tissue layers was modeled in an *in vitro* setting, it was found to diffuse through a multi-cell layer in a peri-cellular manner (Kuiper et al., 2014c). Based on the diffusion parameters and on the measured kinetics of uptake into the cells, the likely distribution of ascorbate in a tumor, at variable plasma concentrations, was modeled. These data indicated that, when plasma concentrations are very low, there is virtually no ascorbate in the tissues and only those cells in the immediate vicinity of the blood vessel are able to accumulate any ascorbate (**Figure 3**). This scenario, modeled for 10 μM plasma levels, represents a state approaching scurvy. When plasma concentrations are 50 μM, a level considered in the healthy range but below plasma saturation (Carr and Frei, 1999; Levine et al., 2001), low levels of ascorbate penetrate up to 100 μm from the blood vessel, but cellular accumulation to physiological low mM concentrations is only seen in the cells immediately adjacent to the blood vessel (**Figure 3**). Optimal intracellular levels could be achieved when plasma is saturated (100 μM), but the diffusion zone does not extend beyond 100 μm. The inference is that 100 μM plasma ascorbate, which is the maximum achievable by oral intake, would not be sufficient to ensure effective distribution to the cells throughout a poorly perfused tumor. When plasma levels were increased to the mM range, the model indicated optimal uptake of cells even at 200 μm from the blood vessel. This information supports the rationale for a number of the hypotheses discussed in this review that indicate an advantage of intravenous ascorbate infusions over oral intake.

## Summary

Recent discoveries have expanded our knowledge of the biological functions of ascorbate, and have highlighted a number of interesting hypotheses that suggest there is a good rationale to investigate the feasibility of using ascorbate as an adjunct treatment for cancer. We believe that the research progress described in this review will soon lead to the development of effective protocols for the use of ascorbate in the cancer clinical setting. Clinical studies underway will help identify which patients might benefit from ascorbate treatment. Given its lack of toxicity, ready availability and low cost, it is our hope that good information on the mechanism(s) of action will aid translation of the new information into clinical practice.

## Author Contributions

MV and AD reviewed the manuscript. MV contributed two-thirds of the writing and formulated the proposal. AD was largely responsible for the sections on epigenetics and other co-factor activities of ascorbate.

## Conflict of Interest Statement

The authors declare that the research was conducted in the absence of any commercial or financial relationships that could be construed as a potential conflict of interest.

## Abbreviations

2-OGDD: 2-oxoglutarate-dependent dioxygenase
AML: acute myeloid leukemia
DHA: dehydroascorbate
GLUT: glucose transporter
HIF: hypoxia-inducible factor
HSC: hematopoietic stem cell
IDH: isocitrate dehydrogenase
JMJC: Jumonji-C-domain-containing
SVCT: sodium dependent vitamin C transporter
